# Increasing throughput of manual microscopy of cell suspensions using solid medium pads

**DOI:** 10.1016/j.mex.2019.02.010

**Published:** 2019-02-15

**Authors:** Alexander I. Alexandrov, Alexander A. Dergalev

**Affiliations:** a Bach Institute of Biochemistry, Research Center of Biotechnology of the Russian Academy of Sciences, 33, bld. 2 Leninsky Ave., Moscow 119071, Russia; b Belozersky Institute of Physico-Chemical Biology, Lomonosov Moscow State University, Moscow, 119234, Russia

**Keywords:** High throughput manual microscopy using solid-medium pads, Microscopy, Yeast, Microbe, Solid medium, High-throughput, Agar, Immobilization

## Abstract

Microscopy of multiple samples using slides and coverslips is time consuming and good images are sometimes difficult to obtain due to cell movement. Our method involves manual spotting of multiple cell samples onto solid-medium pads, which creates the following benefits:

• Rapid, high-quality imaging of multiple samples (hundreds per day) by visible and fluorescence microscopy.
• No need for expensive automated equipment, multi-well plates or large amounts of consumables.
• Wide range of working cell concentrations.

The method was implemented for *S. cerevisiae* yeast, however it is likely to be applicable to all types of microorganisms and possibly other microscopic samples such as pollen. The lack of need for automated equipment may also make this method useful for field work.

## Graphical abstract

**Figure:**
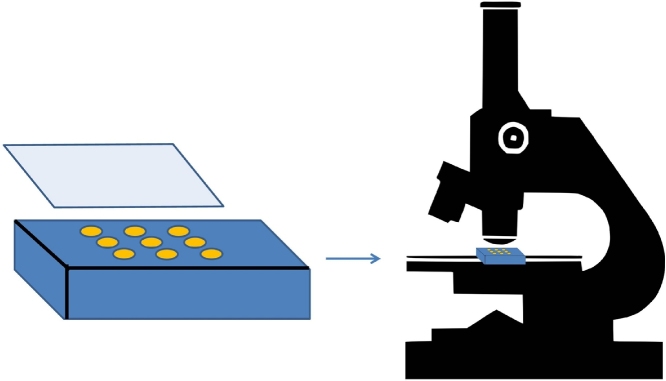

Specifications table

**Table:** 

Subject Area	• Biochemistry, Genetics and Molecular Biology
More specific subject area:	Cell biology, Microscopy
Method name:	High throughput manual microscopy using solid-medium pads
Name and reference of original method	
Resource availability	

## Method

Microscopy is the cornerstone of cell biology and manual, non-automated microscopes are probably the most frequently used tool for imaging, even for multiple samples, due to the high price of motorized equipment suitable for high-throughput studies. Common procedures which require microscopy of numerous samples are screening for transformants expressing fluorescent proteins, or assaying the effects of mutations on a microscopically observable phenotype. The standard method for low-throughput microscopy is to place a drop of cell suspension between a glass slide and a coverslip. This is a simple procedure, however in case of multiple samples, the microscope needs to be refocused for each new slide, and lots of glass and, at high magnifications, immersion oil are expended. Cell movement in suspensions also causes problems, which, at least for yeast samples, is usually prevented by using lectins or poly-lysine to immobilize cells on glass. However, the glass treatment is time-consuming and expensive.

Imaging on thin solid medium pads is commonly used for time-lapse experiments that require cell growth [[1], [2], [3], [4]] and has been used for high-throughput image-based screening [5] by using robots to spot arrays of samples. However, our experience demonstrates the great utility of solid medium pads for imaging multiple samples using regular non-automated microscopy. We provide an approach that enables rapid, high-quality imaging of large numbers of samples using standard optical and fluorescence microscopy on either upright or inverted microscopes, without the need for motorized equipment or lengthy sample preparation. The approach can easily be adopted by any group with access to a microscope to improve the throughput of their work.

**Fig. 1:**
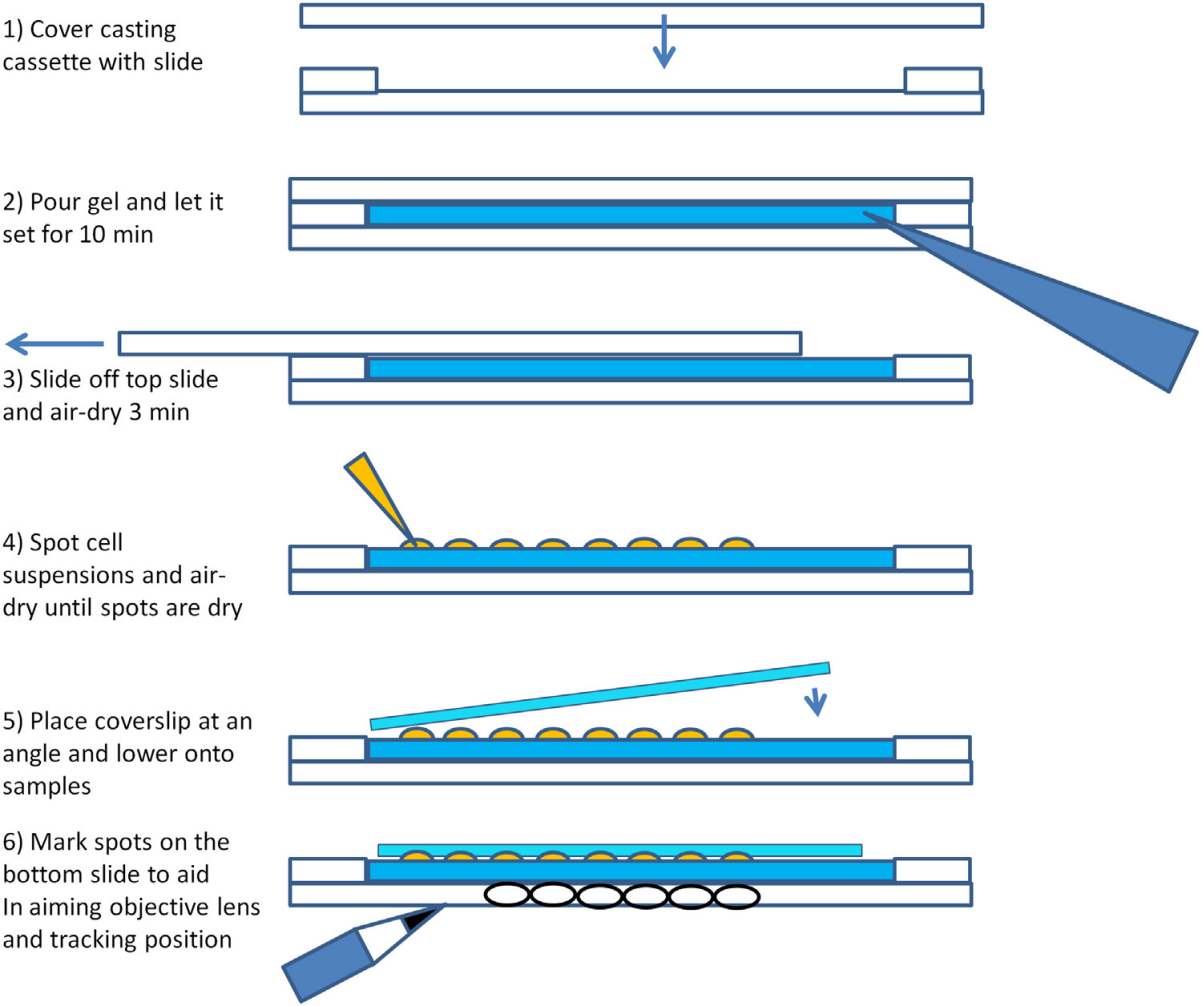
**Preparation of the solid medium pad using the casting cassete.** Schematic of the gel preparation procedure. Numbering of the steps is identical to the description in the main text.

Solid medium pads were prepared using standard glass slides with glued on (cyanacrylate glue) double spacers made from the same slides by cutting them into thin rectangles using a rotary stone-cutting wheel. A similar cassette can be made by using a glass cutter to make spacers and simply taping the spacers to the glass slide with narrow adhesive tape. Solid medium pads can also be cast between glass plates for electrophoresis as long as thicker spacers are used to make gels thicker for increased robustness. Fig. 1 depicts the casting cassete and a graphical protocol for the gel casting and sample application. These involve the following steps::

1) Cover the casting cassette with another glass slide.
2) Pour any melted solid medium between the two slides at the step area. A Pasteur pipette with a 200 μl tip put onto the end is a convenient way to apply the medium between the glasses, however the tip should be put on after the medium is inside pipette. Putting a piece of paper under the casting assembly helps keep the work surface clean of medium spills. If there are no special requirements, we use 2–3% agar in distilled water. Leave to set for ˜5–10 min.
3) After the gel has set, the top glass is carefully removed by sliding it off to the side. This leaves an ideal smooth gel surface. **Important:** Air-dry the solid medium pad for a few minutes to prevent sample droplet mixing.
4) Spot cell suspensions onto the medium pad using a wire loop or a pipette. Up to 36 spots of samples can be fit under a standard 24 x 24 mm coverslip. Material from a colonies in a dish can also be directly streaked onto the pad. Let the sample spots dry (1–3 min).

(Optional) If using an inverted microscope, cut out the section of gel containing your sample with a clean coverslip and use it to place the gel face down onto a glass bottom dish. Cover the back side of the gel with a cover glass and proceed to step 6

1) Cover the samples with a coverslip by lowering it from one side, this helps prevent air bubbles.
2) Turn over the slide and mark your cell suspension areas with a black marker (circles or dots are fine). This will help aiming the objective at the sample spot.
3) Once the slide is fixed in the microscope, aim the objective at the first spot in your array and focus. **Important!** Keep track of your position on the array.

**Fig. 2:**
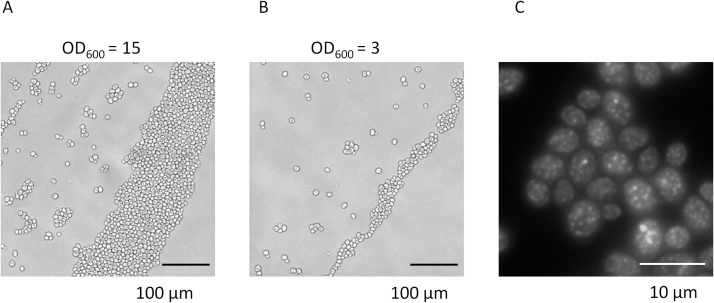
**Representative images obtained using the solid agar pad**. Cells of the BY4741 Ssa1-GFP strain [6] (Mat**a** his3Δ1 leu2Δ0 met15Δ0 ura3Δ0 Ssa1-GFP::HIS3(S. pombe his5)) were spotted onto 3% agar at different densities (A, B) or 2% agar containing 1 M potassium chloride (C), which causes formation of small foci (article in preparation). Imaging was performed using an inverted Invitrogen FLoid (20x NA 0.45 objective lens) for (A) and (B), and an upright Zeiss AxioSkop 40 with a 100x NA 1.2 oil immersion objective lens for (C).

Images obtained using this method are presented in Fig. 2. Multiple samples can be imaged very rapidly, since there is no need to exchange slides and virtually no cell movement, at least for yeast cells. Transition between each sample is very quick because all the samples are nearly in the same focal plane. In our hands, imaging up to 36 samples takes ˜1-2 h, depending on various factors such as exposition times, z-stacking, etc. Viewing without imaging can be done considerably faster. Yeast cells form a monolayer in a wide range of concentrations (OD_600_ from 1 to 20 are optimal) and cell density is always higher near the edges of the drop. This is beneficial if numerous cell images are required for quantification. Less dense areas will also be available in the middle of the spot if needed.

Currently we always use this approach instead of the standard slide and coverslip method if we plan on collecting images of even a few samples. It can be used both on upright and inverted microscopes. The gel casting process takes around 5–10 min and gels can be used throughout the day for numerous samples by simply taking off the coverslip, spotting new samples onto empty areas and then covering the gel with a new coverslip. A medium pad that is in use can be stored for hours by covering the pad surface with coverslips to minimize drying and placing it in a refrigerator. Also, cassettes with pads trapped between slides can be made in advance, wrapped in plastic and stored for days at 4 °C.
